# Mechanics-based classification rule for plants

**DOI:** 10.1073/pnas.2308319120

**Published:** 2023-10-06

**Authors:** Tohya Kanahama, Motohiro Sato

**Affiliations:** ^a^Graduate School of Engineering, Hokkaido University, Sapporo 060-8628, Japan; ^b^Faculty of Engineering, Hokkaido University, Sapporo 060-8628, Japan

**Keywords:** plant, turgor pressure, geometric rigidity, scaling law, classification rule

## Abstract

The height of thick and solid plants, such as woody plants, is proportional to two-thirds of the power of their diameter at breast height. However, this rule cannot be applied to herbaceous plants that are thin and soft because the mechanisms supporting their bodies are fundamentally different. This study aims to clarify the rigidity control mechanism resulting from turgor pressure caused by internal water in herbaceous plants to formulate the corresponding scaling law. We modeled a herbaceous plant as a cantilever with the ground side as a fixed end, and the greatest height was formulated considering the axial tension force from the turgor pressure. The scaling law describing the relationship between the height and diameter in terms of the turgor pressure was theoretically derived. Moreover, we proposed a plant classification rule based on stress distribution.

## Significance

Herbaceous plants, which are thin and soft, support their bodies using the turgor pressure caused by internal water. The results of this research promote a correct understanding of geometric rigidity due to tension caused by turgor pressure in the field of botany. Furthermore, this research is expected to serve as a starting point for opening up an interdisciplinary research field that links mechanical theory in engineering with the vast amount of measured and observed data in botany.

The height of a woody plant is proportional to two-thirds of the power of its diameter at breast height (1–10). Although trees exhibit extremely diverse forms with dissimilar shapes, this law proposed by Greenhill in 1881 is broadly applicable. Based on the theory of structural mechanics, Greenhill derived the greatest height at which buckling occurs owing to the self-weight of a cantilever of circular cross-section and constant diameter in the direction of height, expressed as follows:

[1] $$L_{c}={\left(C\frac{E}{\gamma }r_{l}^{2}\right)}^{1/3},$$

where $L_{c}$   denotes the greatest height [m], E   indicates the elastic modulus [N/m^2^], γ   represents unit volume weight [N/m^3^], $r_{l}$   denotes the radius in a fixed end [m], and C   is a constant ( C≈1.959   ). This formula indicates that the greatest height of a solid cylinder against self-weight buckling is proportional to two-thirds of the power of its diameter (1), and McMahon demonstrated that the scaling law of height–diameter based on Greenhill’s theory is applicable to actual trees as well (2, 3). Through these verification results, Greenhill’s achievements have been widely applied in the fields of ecology, forest science, and engineering (5–24).

Nonetheless, this scaling law is suitable for only solid and large woody plants such as trees and is invalid for soft and small herbaceous plants (20–24). The differences in the body-support mechanism between woody and herbaceous plants must be considered.

Woody plants can resist gravity by their own bending rigidity owing to their sufficiently large and rigid trunks (5, 6, 8, 25, 26). In contrast, herbaceous plants are slender and in most cases have hollow cross-sections. Moreover, their bending rigidity is considerably lower than that of woody plants (20, 27–29). Thus, herbaceous plants tend to support their own bodies by leveraging the turgor pressure induced by internal moisture (29–34).

**Fig. 1.**
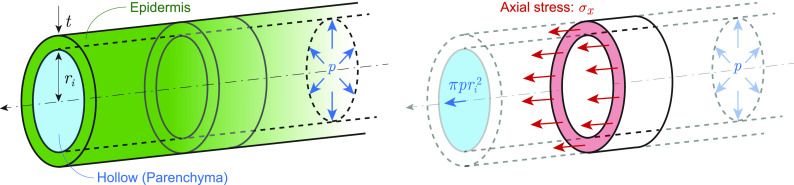
Tension force resulting from turgor pressure. This model is cylindrical with inner radius $r_{i}$ and epidermis thickness t [m] (Fig. 1). From the equilibrium of forces in the axial direction, we obtain the relationship $T=\pi pr_{i}^{2}$ [N] [i.e., the axial stress $\sigma_{x}=T/2\pi r_{i}t=pr_{i}/2t$ (N/m^2^)] between the axial tension force T [N] and turgor pressure p [N/m^2^]. This force generates the geometric rigidity that differs from the flexural rigidity.

Because the flexural rigidity of the dry-state parenchyma is substantially lower than that of the epidermis in herbaceous plants, the plant stem can be modeled as a thin cylinder with an inner radius $r_{i}$   and epidermis thickness t   [m] (Fig. 1). In this model, if the amount of water inflow exceeds that of water outflow, turgor pressure p   [N/m^2^] is generated to balance the internal pressure, resulting in tension along the axial direction $T=\pi pr_{i}^{2}$   [N] and inducing geometric rigidity that improves the rigidity against out-of-plane loads. This is analogous to the phenomenon in which the pitch of a musical instrument increases upon stretching the strings (35–37). Accordingly, by this function, herbaceous plants can support self-weight, despite their soft and slender bodies. However, most previous studies related to plant morphology misinterpreted this body-support mechanism. They either ignored the tension-induced geometric rigidity (20, 21, 23) or confused it with the flexural rigidity of the material (38). For example, the elastic modulus of stems obtained from the deflection caused by a three-point flexural test cannot be compared with the equation based on the beam theory without considering the tension force by the turgor pressure.

This study aims to derive theoretically the scaling law in terms of turgor pressure and consolidate the mechanics’ theory of the plant height based on geometric rigidity by clarifying the effect of the axial tension induced by the turgor pressure on the greatest height of herbaceous plants. This will define the differences between the geometric rigidity induced by the tension force and the flexural rigidity of the material and thus is expected to aid the development of future research focused on plant morphology.

## Methods

**Fig. 2.**
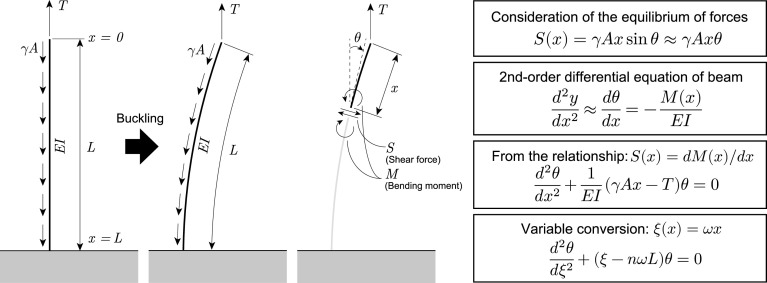
Calculation model and formulation flow. This model is a cylindrical cantilever with cross-sectional area A [m^2^], unit volume weight γ [N/m^3^], flexural rigidity EI [Nm^2^], and length L [m]. From the equilibrium of forces in the self-buckling state and the relationship between shear force S [N] and bending moment M [Nm] at position x from tip, we obtain the governing equation. In this study, we changed the governing equation using variable conversion ( ξ=ωx).

Herein, the calculation model considered a cantilever subjected to its self-weight q [N/m] and vertical tension force T [N] generated by the turgor pressure (Fig. 2). The coordinate system was defined as x=0 at the fixed end and x=L at the free end; the bending rigidity EI [N·m^2^] and the cross-sectional area A [m^2^] are assumed to be constant in the axial direction. The plant model was a horizontally oriented thin-walled cylinder with closed ends enclosing a cavity filled with water.

We considered the case in which the vertical tension force T is applied at the top of the cantilever deflected by its own self-weight. Accordingly, considering the equilibrium of the forces at any point, if the deflection y [m] can be assumed as negligibly small, the general equation for the deflection angle θ can be obtained as follows:

[2] $$\frac{d^{2}\theta }{d\xi^{2}}+\left(\xi -n\omega L\right)\theta =0.$$

This equation conducted the variable conversion for position x using ξ=ωx ; the variable conversion parameter ω is expressed as

[3] $$\omega ={\left(\frac{\gamma A}{\mathrm{EI}}\right)}^{1/3}.$$

Moreover, n of Eq. **3** is a dimensionless parameter representing the ratio of tension force T to the volume force by self-weight ( n=γAL).

The general solution of Eq. **2** includes two arbitrary constants that were derived by applying the boundary condition of a cantilever at a free end to it (bending moment $M\left(0\right)=d\theta /dx$ = 0 at x=0 ), among which one constant can be deleted. By applying the boundary condition of the cantilever at the fixed end to it (deflection angle $\theta \left(L_{c}\right)=0$ at $x=L_{c}$ ), if another constant was assumed nonzero for obtaining a significant solution, the Eigen equation regarding $\xi_{c}$ for calculating the greatest height $L_{c}$ can be derived as follows:

[4] $$Re\left(Ai\left(\Xi_{c}\left(\xi_{c}\right)\right){Bi}^{'}\left(\Xi_{c}\left(\xi_{c}\right)-{\left(-1\right)}^{1/3}\xi_{c}\right)-{Ai}^{'}\left(\Xi_{c}\left(\xi_{c}\right)-{\left(-1\right)}^{1/3}\xi_{c}\right)Bi\left(\Xi (\xi_{c})\right)\right)=0,$$

where $Ai\left(x\right)$ denotes the first type of Airy function, $Bi\left(x\right)$ represents the second type of Airy function, and prime indicates the first differentiation associated with ξ . Moreover, Ξ(ξ) can be expressed as follows:

[5] $$\Xi_{c}\left(\xi_{c}\right)={\left(-1\right)}^{1/3}\left(1-n\right)\xi_{c}.$$

Consequently, as Eq. **4** includes two parameters, ξ   and n   , the solution $\xi_{c}$   of Eq. **4** is a function of only the tension parameter n   . Based on Eq. **4**, which is derived by applying the boundary condition at the fixed end, Eq. **3**, and the relation of variable conversion ξ=ωx , the greatest height $L_{c}$ was calculated considering the tension force against the self-weight buckling in a hollow plant, as depicted in Fig. 1.

[6] $$L_{c}={\xi_{c}\left(n\right)\left(\frac{E}{4\gamma }\left(1+\alpha^{2}\right)r_{l}^{2}\right)}^{1/3}=\;{\left(1+\alpha^{2}\right)}^{1/3}{\frac{\xi_{c}\left(n\right)}{\xi_{c}\left(0\right)}\left(C\frac{E}{\gamma }r_{l}^{2}\right)}^{1/3},$$

where α   indicates the ratio of inner radius $r_{i}$   to outer radius $r_{o}$   ( $\alpha =r_{i}/r_{o}$   ; α=0   in solid cross-section). As indicated by Eq. **6**, the scaling law between the height and diameter was consistent even if the influence of tip force is considered. Moreover, Eq. **6** can be applicable to compressive force.

As Eq. **4** can be strictly solved, the solution was obtained using numerical calculations with the secant method. Regarding the eigenvalue $\xi_{c}$   satisfying Eq. **4**, the numerical solution was obtained using the discrete tension parameter Δn=-3.00,-2.99,⋯,1.00 . By using calculation software such as Mathematica 12.1, when the relative error of the solution on repetition count ( m-1 ) from a solution on repetition count m is less than $1.0\times 10^{-5}$ , we deemed it as convergence.

## Results and Discussion

**Fig. 3.**
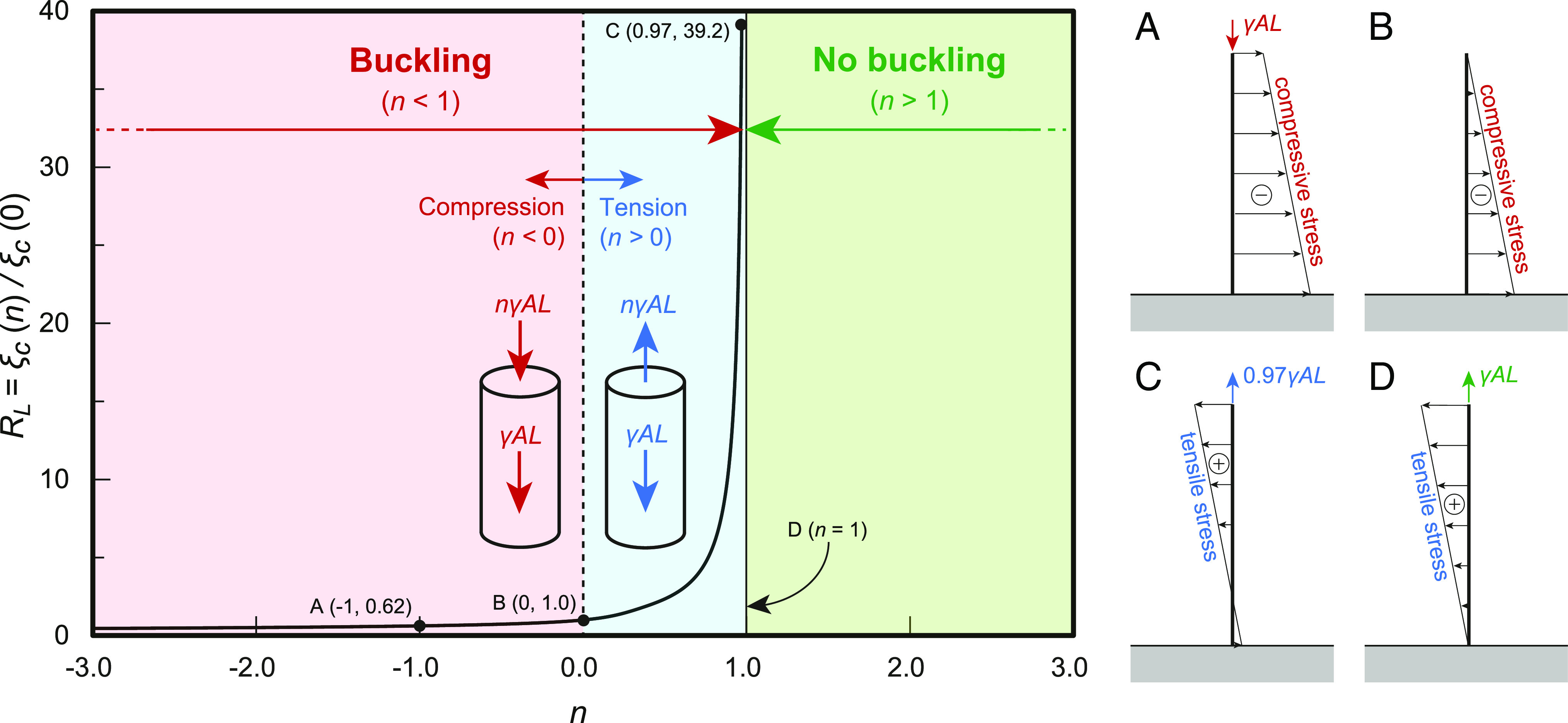
Effect of the tip force on the greatest height and axial stress distributions. *R_L_* is the ratio of the greatest height with a tension force to that without a force [ $R_{L}=\xi_{c}\left(n\right)/\xi_{c}\left(0\right)$ ], and *n* is a tension parameter (the ratio of the tip force to the total self-weight). The red and blue area represents the region about the occurrence of buckling. In contrast, the green area is the no-buckling region. On right-hand side of this figure, the stress distributions at points (*A*–C), and line (*D*) in this figure are shown.

The relationship between the tension parameter n   and the greatest height ratio $R_{L}$   is portrayed in Fig. 3, including the axial stress distribution on points A, B, and C and line D. In both cases, the vertical axis represents the ratio of the greatest height with the tension force to that without the force [ $R_{L}=\xi_{c}\left(n\right)/\xi_{c}\left(0\right)$ ], and the horizontal axis represents the tension parameter n.

In case of no tip force, i.e., n=0 , from the greatest height ratio $R_{L}=1$ , the consistency can be validated with Greenhill’s formula Eq. **1**. The greatest height ratio $R_{L}$ increased with n and vice versa.

The results shown in Fig. 3 indicate that when a compressive force acts at the top ( n<0 ), the greatest height decreases with increasing load, and under a compressive force of magnitude equal to the volumetric force ( n=1 ), the greatest height decreases by approximately 40%. The gradual reduction in the greatest height with the decreasing tension parameter was expected to be caused by the reduction in the absolute value of the load with the decreasing greatest height. This result of the compressive load may be applied to the estimation of the greatest height considering the crown weight of the tree.

In contrast, when a tension force is applied at the top, the greatest height increases with the load, and the greatest height becomes approximately 40 times higher at n=0.97, compared to the case of n=0 . As discussed earlier, the greatest height tends to increase monotonically as the tension parameter *n* increases. In the range of *n* > 0.97, ξ satisfying the eigen equation in Eq. **4** cannot be numerically obtained, regardless of setting the initial value of the secant method along with the increment width. This can be attributed to the axial stress distribution comprising only tensile stress throughout the span in n≥1.

First, if the axial compressive force at the tip is equal to the self-weight ( n=-1 , point A) and no axial tension force is applied at the tip ( n=0 , point B) in the current calculation model, only the compressive force caused by the self-weight acts throughout the cross-section, resulting in a stress distribution that causes only compressive stress. Thereafter, for the maximum tension parameter ( n=0.97 , point C), the axial tension at the tip is almost equal in magnitude to the volume force γAL , generating tensile stress at most locations over the entire span. Nonetheless, an exceedingly small amount of compressive stress was generated near the fixed end. In the case of a tension force T of magnitude precisely equal to the volume force γAL ( n=1 , Line D), the compressive stress disappears completely, and only tensile stress is generated over the entire span.

As such, buckling is an instability problem caused by compressive forces. In the case of n≥1 , where only tensile stresses occur over the entire span, buckling does not occur, and consequently, ξ satisfying the eigen equations in Eq. **4** disappears. Therefore, from the perspective of structural mechanics, the greatest height of a plant that satisfies n≥1 is no longer constrained by the self-weight buckling.

Thus, by the definition of the tension parameter n [ratio of tension force T to the volume force by self-weight ( n=γAL )] and the relationship between the turgor pressure p and tension force T ( $T=\pi pr_{i}^{2}$ ), we can derive the equation for assessing whether the greatest height is constrained by self-buckling as follows:

[7] $$n=R_{s}R_{c}\geq 1,$$

where $R_{s}$ and $R_{c}$ represent the dimensionless parameters as follows:

[8] $$R_{s}=\frac{p}{\gamma L},\;R_{c}=\frac{\alpha^{2}}{1-\alpha^{2}},$$

$R_{s}$   denotes the ratio of turgor pressure p   to the maximum stress $\sigma_{max}=\gamma AL/A$   at the fixed end by self-weight, and $R_{c}$   indicates the degree of the hollowness of the cross-section. When Eq. **7** is satisfied, tension prevails over the entire cross-section, and no buckling occurs under self-weight. In particular, Eq. **7** indicates whether bending rigidity or geometric rigidity is dominant. Based on the viewpoint of mechanical and structural support mechanisms and using Eq. **7**, plants can be classified into two main categories. Although Eq. **7** does not consider the tapered shape exhibited by several plants, if only tensile stresses are applied over the entire span in tapered shapes, theoretically, the occurrence of self-weight buckling should be avoided.

We also considered the mechanical and geometrical properties of woody and herbaceous plants. Most woody plants have solid, heavy, thick, and large bodies (5, 6, 8, 25, 26, 39). These characteristics reduce the tension parameter n   . Therefore, the condition in Eq. **7** is not satisfied. In contrast, most herbaceous plants exhibit hollow, light, thin, and small bodies (20, 27–29). This characteristic increases the tension parameter n   . Therefore, the condition in Eq. **7** is satisfied for herbaceous plants, and deadweight buckling is avoided. This theoretically supports the fact that Greenhill’s scaling law does not apply to herbaceous plants, as reported by Niklas (20, 21) and Norberg (24).

Moreover, from the relationship $n=R_{s}R_{c}$ , the following equation can be obtained:

[9] $$L_{c}=\frac{1}{n}\frac{p}{\gamma }R_{c}.$$

When Eq. **7** is satisfied, the greatest height is defined by Eq. **9** rather than by Eq. **6**. Eq. **9** is derived under the condition that the maximum compression force caused by the volume force is equal to the tension force caused by the turgor pressure. Consequently, Eq. **9** does not include elastic modulus E and moment of inertia I . It is conceivable that Eq. **9** represents a state in which the geometric rigidity distinguishes. Moreover, as the tension parameter n is present in the denominator, increasing the tension force to self-weight is not substantially effective for acquiring the greatest height. This is because Eq. **9** expresses the relation of the greatest height based on stress.

**Fig. 4.**
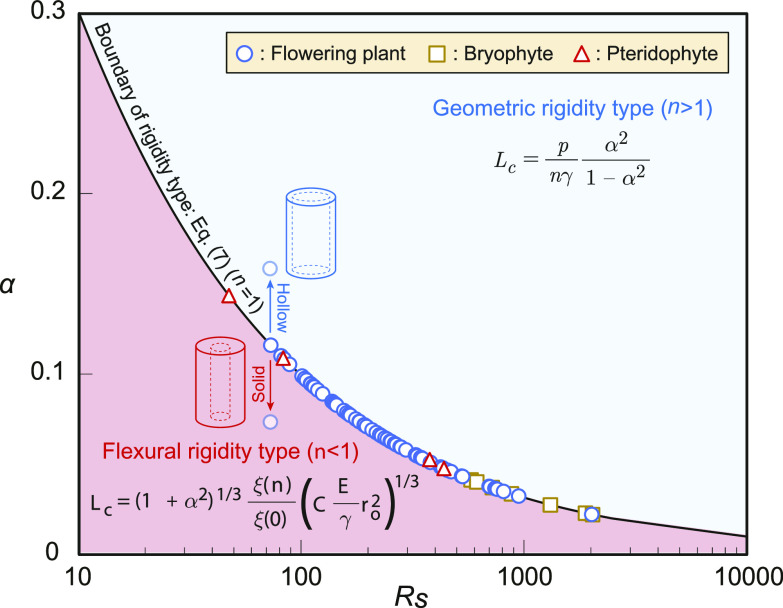
Classification of herbaceous plants based on the rigidity type. Ratio *Rs* of the turgor pressure to the maximum stress was calculated using the values of height *L* and unit volume weight *γ* measured by Niklas (23) for 76 herbaceous plants species [⚪: flowering plant (65 species), ◻: bryophyte (seven species), △: pteridophyte (four species)]. From these results, we obtained the hollow ratio when parameter *n* = 1 (i.e., the markers indicate the lowest hollow ratio α that sustains the geometric rigidity type). The solid line represents the results obtained by solving Eq. **7** for α with *n* = 1.

The calculation results of hollow ratio α   indicating the ratio of inner radius $r_{i}$   to outer radius $r_{o}$   for parameter n=1   in Eq. **7** are illustrated in Fig. 4. These are based on the values of height L   and unit volume weight γ   measured by Niklas (23) for 76 herbaceous plants species [⚪: Flowering plant (65 species), ◻: Bryophyte (seven species), △: Pteridophyte (four species)]. The vertical axis represents hollow ratio α   , and the horizontal axis represents $R_{s}$   , the ratio of the turgor pressure to the maximum stress; the solid line represents the result obtained by solving Eq. **7** for α   with n=1   (*i.e.*, the markers indicate the lowest hollow ratio α that sustains the geometric rigidity type).

Therefore, for the blue area located above the solid line, the plant morphology avoids buckling under self-weight, indicating that the body is primarily supported by geometric rigidity. For the red area located below the solid line, the bending rigidity is dominant, and Eq. **6** can be considered applicable as a scaling law. For all 76 types, a lower limit of P = 0.3 MPa was used for swelling pressure P in the calculations, from the general range of P = 0.3 to 1.0 MPa reported in a previous study (28). If the plants become hollower, each point moves up; and if they become more solid, each point moves down.

Based on the results shown in Fig. 4, for example, for the hollow ratio α=0.1 in all plants, few species were associated with the mode supporting the body by flexural rigidity (red area). In contrast, for α≥0.15 , all 76 species exhibited a body support mode owing to geometric rigidity (blue area). If all the plants measured by Niklas can be modeled as thin cylinders (Fig. 1) from the range of hollow ratio α in real plants (40), all of them should satisfy Eq. **7**. Therefore, actual herbaceous plants avoid self-weight buckling and support their bodies with geometric rigidity.

Moreover, we can presume that trees with an almost solid cross-section ( $R_{c}\approx 0$ ) and bamboos that are hollow but not saturated with water ( $R_{s}\approx 0$ ) in the cavity pertain to the range of n≤1 . Thus, they may conceivably support their body via bending rigidity. Consequently, Greenhill’s law can be applied to these species.

Thus, Eq. **6** derived in this study can be used to classify plants based on mechanical and structural support mechanisms involving their physiology, in contrast to the existing ecologically highly detailed plant classification. This can be applied as a general rule. However, its applicability requires further verification by actual measurement and statistical approaches, including further examination of the validity of the proposed model for the applicable plants.

## Data Availability

All study data are included in the main text.

## References

[1] A. G. Greenhill, Determination of the greatest height consistent with stability that a vertical pole or mast can be made, and the greatest height to which a tree of given proportions can grow. Proc. Camb. Philos. Soc. **4**, 62–73 (1881).

[2] T. McMahon, Size and shape in biology: Elastic criteria impose limits on biological proportions, and consequently on metabolic rates. Science **179**, 1201–1204 (1973). 4689015 10.1126/science.179.4079.1201

[3] T. A. McMahon, R. E. Kronauer, Tree structures: Deducing the principle of mechanical design. J. Theor. Biol. **59**, 443–466 (1976). 957700 10.1016/0022-5193(76)90182-x

[4] T. von Karman, M. A. Biot, Mathematical Methods in Engineering (Cambridge University Press, Cambridge, 1940).

[5] J. A. Adam, Mathematics in Nature: Modeling Patterns in the Natural World (Princeton University Press, Princeton, 2003).

[6] D. King, O. L. Loucks, The theory of tree bole and branch form. Radiat. Environ. Biophys. **15**, 141–165 (1978). 724980 10.1007/BF01323263

[7] K. J. Niklas, Plant Biomechanics: An Engineering Approach to Plant Form and Function (The University of Chicago Press, Chicago, 1992).

[8] K. J. Niklas, Plant Allometory: The Scaling of Form and Process (The University of Chicago Press, Chicago, 1994).

[9] K. J. Niklas, Maximum plant height and the biophysical factors that limit it. Tree Physiol. **27**, 433–440 (2007). 17241985 10.1093/treephys/27.3.433

[10] S. T. O’Brien, S. P. Hubbell, P. Spiro, R. Condit, R. B. Foster, Diameter, height, crown, and age relationship in eight neotropical tree species. Ecology **76**, 1926–1939 (1995).

[11] T. Kanahama, M. Sato, Mathematical modelling to determine the greatest height of trees. Sci. Rep. **12**, 2039 (2022). 35132088 10.1038/s41598-022-06041-w PMC8821560

[12] T. Kanahama, T. Fujimura, M. Sato, Critical height for self-weight buckling in tapered trees. J. Jpn. Soc. Civil Eng. Ser. **77**, 62–71 (2021).

[13] T. Kanahama, M. Sato, Summation rules in critical self-buckling states of cylinders. Mech. Res. Commun. **123**, 103905 (2022).

[14] N. M. Holbrook, F. E. Putz, Influence of neighbors on tree form: Effects of lateral shade and prevention of sway on the allometry of *Liquidambar styraciflua* (sweet gum). Amer. J. Bot. **76**, 1740–1749 (1989).

[15] C. Goudenhooft, T. Almeras, A. Bourmaud, C. Baley, The remarkable slenderness of flax plant and pertinent factors affecting its mechanical stability. Biosyst. Eng. **178**, 1–8 (2019).

[16] M. Aiba, T. Nakasizuka, Differences in the dry-mass cost of sapling vertical growth among 56 woody species co-occurring in a Bornean tropical rain forest. Funct. Ecol. **21**, 41–49 (2007).

[17] T. D. Jackson , The mechanical stability of the world’s tallest broadleaf trees. Biotropica **53**, 110–120 (2021).

[18] M. Fournier, J. Dlouha, G. Jaouen, T. Almeras, Integrative biomechanics for tree ecology: Beyond wood density and strength. J. Exp. Bot. **64**, 4793–4815 (2013). 24014867 10.1093/jxb/ert279

[19] M. Franco, C. K. Kelly, The interspecific mass-density relationship and plant geometry. Proc. Natl. Acad. Sci. U.S.A. **95**, 7830–7835 (1998). 9636236 10.1073/pnas.95.13.7830 PMC22773

[20] K. J. Niklas, The scaling of plant height: A comparison among major plant clades and anatomical grades. Ann. Bot. **72**, 165–172 (1993).

[21] K. J. Niklas, Influence of tissue density-specific mechanical properties on the scaling of plant height. Ann. Bot. **72**, 173–179 (1993).

[22] K. J. Niklas, H.-C. Spatz, Growth and hydraulic (not mechanical) constraints govern the scaling of tree height and mass. Proc. Natl. Acad. Sci. U.S.A. **101**, 15661–15663 (2004). 15505224 10.1073/pnas.0405857101 PMC524850

[23] K. J. Niklas, Plant height and the properties of some herbaceous stems. Ann. Bot. **75**, 133–142 (1995).

[24] R. A. Norberg, Theory of growth geometry of plants and self-thinning of plant populations: Geometric similarity, elastic similarity, and different growth modes of plant parts. Am. Nat. **131**, 220–256 (1988).

[25] R. Gonçalves, G. H. L. Garcia, S. Brazolin, C. Bertoldo, M. Ruy, Methodology for the characterization of elastic constants of wood from tree branches. BioResources **14**, 8439–8454 (2019).

[26] K. J. Niklas, Mechanical properties of black locust (*Robinia pseudoacacia* L.) wood. Size- and age-dependent variations in sap- and heartwood. Ann. Bot. **79**, 265–272 (1997).

[27] M. E. Olson, R. Aguirre-Hernandez, J. A. Rosell, Universal foliage-stem scaling across environments and species in dicot trees: Plasticity, biomechanics and Corner’s Rules. Ecol. Lett. **12**, 210–219 (2009). 19141123 10.1111/j.1461-0248.2008.01275.x

[28] C. Wei, P. M. Lintilhac, Loss of stability: A new look at the physics of cell wall behavior during plant cell growth. Plant Physiol. **145**, 763–772 (2007). 17905864 10.1104/pp.107.101964 PMC2048773

[29] W. Li , Protocol for mapping the variability in cell wall mechanical bending behavior in living leaf pavement cells. Plant Physiol. **188**, 1435–1449 (2022). 34908122 10.1093/plphys/kiab588 PMC8896622

[30] D. J. Cosgrove, Wall extensibility: Its nature, measurement and relationship to plant cell growth. New Phytol. **124**, 1–23 (1993). 11537718 10.1111/j.1469-8137.1993.tb03795.x

[31] C. Wei, P. M. Lintilhac, J. J. Tanguay, An insight into cell elasticity and load-bearing ability. Measurement and theory. Plant Physiol. **126**, 1129–1138 (2001). 11457963 10.1104/pp.126.3.1129 PMC116469

[32] L. Zonia, T. Munnik, Life under pressure: Hydrostatic pressure in cell growth and function. Trends Plant Sci. **12**, 90–97 (2007). 17293155 10.1016/j.tplants.2007.01.006

[33] S. Tsugawa , Elastic shell theory for plant cell wall stiffness reveals contributions of cell wall elasticity and turgor pressure in AFM measurement. Sci. Rep. **12**, 13044 (2022). 35915101 10.1038/s41598-022-16880-2 PMC9343428

[34] M. Asaoka , Stem integrity in *Arabidopsis thaliana* requires a load-bearing epidermis. Development **148**, dev198028 (2021). 33637612 10.1242/dev.198028

[35] M. Saitoh, A. Okada, The role of string in hybrid string structure. Eng. Struct. **21**, 756–769 (1999).

[36] I. Handžić, K. B. Reed, The musical kinetic shape: A variable tension string instrument. Appl. Acoust. **85**, 143–149 (2014).

[37] A. V. Olver, D. Wilson, P. S. J. Crofton, Investigation of service failures of steel music wire. Eng. Fail. Anal. **14**, 1224–1232 (2007).

[38] D. U. Shah, T. P. S. Reynolds, M. H. Ramage, The strength of plants: theory and experimental methods to measure the mechanical properties of stems. J. Exp. Bot. **68**, 4497–4516 (2016). 10.1093/jxb/erx245 28981787

[39] T. Kanahama, S. Tsugawa, M. Sato, Rigidity control mechanism by turgor pressure in plants. Sci. Rep. **13**, 2063 (2023). 36739460 10.1038/s41598-023-29294-5 PMC9899264

[40] G. N. Karam, L. J. Gibson, Biomimicking of animal quills and plant stems: Natural cylindrical shells with foam cores. Mater. Sci. Eng. C. **2**, 113–132 (1994).

